# Trifurcated Splitting of Water Droplets on Engineered Lithium Niobate Surfaces

**DOI:** 10.1021/acsami.3c16573

**Published:** 2024-01-09

**Authors:** Sebastian Cremaschini, Alberto Cattelan, Davide Ferraro, Daniele Filippi, Filippo Marinello, Alessio Meggiolaro, Matteo Pierno, Cinzia Sada, Annamaria Zaltron, Paolo Umari, Giampaolo Mistura

**Affiliations:** Dipartimento di Fisica e Astronomia “G. Galilei”, Università di Padova, Via Marzolo 8, 35131 Padova, Italy

**Keywords:** optofluidics, lithium niobate, photovoltaic effect, pyroelectric effect, lubricant-infused surfaces

## Abstract

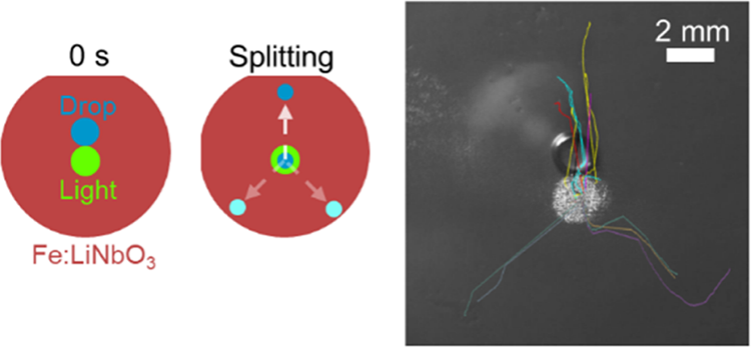

Controlled splitting of liquid droplets is a key function in many microfluidic applications. In recent years, various methodologies have been used to accomplish this task. Here, we present an optofluidic technique based on an engineered surface formed by coating a *z*-cut iron-doped lithium niobate crystal with a lubricant-infused layer, which provides a very slippery surface. Illuminating the crystal with a light spot induces surface charges of opposite signs on the two crystal faces because of the photovoltaic effect. If the light spot is sufficiently intense, millimetric water droplets placed near the illuminated spot split into two charged fragments, one fragment being trapped by the bright spot and the other moving away from it. The latter fragment does not move randomly but rather follows one of three well-defined trajectories separated by 120°, which reflect the anisotropic crystalline structure of Fe:LiNbO_3_. Numerical simulations explain the behavior of water droplets in the framework of the forces induced by the interplay of pyroelectric, piezoelectric, and photovoltaic effects, which originate simultaneously inside the illuminated crystal. Such a synergetic effect can provide a valuable feature in applications that require splitting and coalescence of droplets, such as chemical microreactors and biological encapsulation and screening.

## Introduction

Controlling the mobility of liquid droplets on solid surfaces is a key feature in a variety of applications, ranging from chemical reactions to clinical screening, to name a few.^1−5^ Recently, much attention has been devoted to direct light-driven manipulation of droplets on suitable photoactive surfaces,^6−12^ because light-induced effects can provide contactless, reversible spatial and temporal control without the use of moving parts. Ferroelectric crystals, such as iron-doped lithium niobate, Fe:LiNbO_3_, are promising substrates for this application. If they are locally heated, for instance, by shining infrared light, electric fields are generated by the pyroelectric effect, which are exploited to perform basic microfluidic operations, such as dispensing^13^ and droplet manipulation.^14^ More commonly, they are illuminated with visible light that induces electric charges on their surface due to the photovoltaic effect, generating an internal electric field that can easily reach strengths of hundreds of kV/cm.^15^ The evanescent field, that is, the photoinduced electric field acting outside the illuminated Fe:LiNbO_3_, interacts with neutrally charged micro-objects through dielectrophoretic forces that can be used to actively manipulate them.^16−27^ It should be noted that the term “evanescent” is used in the specialist literature to indicate electric fields that extend outside a globally neutral slab.^20,24−26,28^ More recently, the photovoltaic effect has also been exploited to actuate water droplets on open surfaces.^28,29^ In particular, we have succeeded in the realization of a flexible optofluidic platform based on *z*-cut iron-doped lithium niobate crystals coated with a slippery liquid-infused surface (LIS)^30,31^ that guarantees robust and reliable manipulation of droplets.^32^ These biomimetic surfaces are textured materials infused with a suitable oil. The premise of such a design is that the liquid surface is intrinsically smooth and free from defects down to the molecular scale and can effectively repel immiscible liquids of virtually any surface tension.^31^ They allow easy manipulation of small droplets, for example, tiny water droplets begin to move on the LIS at inclination angles well below 5°. Similarly, droplets of highly viscous polymeric solutions that, on solid surfaces, barely move^33^ or leave traces behind can easily be displaced on the LIS.^34,35^ With our optofluidic platform, sessile water droplets with volumes of microliters, corresponding to millimeters in size, can be easily moved, guided, merged, and split by illuminating a *z*-cut Fe:LiNbO_3_ crystal with an ordinary spatial light modulator. The actuated droplets can cover distances of centimeters in a time scale of less than one second. Similarly, Tang et al. reported the self-propulsion of water droplets in well-defined directions on LiNbO_3_ crystalline surfaces due to the generation of a surface electric potential through thermoelastic–piezoelectric coupling and the pyroelectric effect.^36^

Controlled splitting of droplets is one of the most complicated tasks in droplet manipulation.^37^ Laterally-offset modulated surface acoustic waves^38^ and integrated dielectrowetting devices^39^ have been used to split droplets. Recently, the splitting of droplets on an open microfluidic platform has also been achieved in electrowetting-on-dielectric (EWOD).^37^ In optofluidics, controlled splitting of microdroplets (volume Ω < 0.1 μL) is generally reported by illuminating *y*-cut Fe:LiNbO_3_ crystals,^28,40,41^ where the evanescent field is oriented primarily parallel to the surface of the crystal rather than orthogonally to it, as in a *z*-cut crystal. For example, Puerto et al. investigated aqueous microdroplets hanging at the interface between paraffin oil and air;^28^ in this nonstandard configuration, the evanescent field generated by illuminating the underlying *y*-cut Fe:LiNbO_3_ substrate can also split the water droplets. Li et al. demonstrated an all-optical active mode of oil microdroplet splitting in a sandwich structure consisting of two antisymmetrical *y*-cut Fe:LiNbO_3_ substrates,^40^ while Zhang et al. succeeded in photovoltaic splitting of water microdroplets on a *y*-cut Fe:LiNbO_3_ substrate coated with an LIS.^41^ Although only microdroplets were considered in these works, the main limit of the *y*-cut is that it does not provide a simple way to actuate and guide droplets along well-defined paths. In fact, in lithium niobate, the photovoltaic effect occurs mainly along the *z* axis of the material, leading to light-induced electric fields that are almost an order of magnitude larger in this direction with respect to the *y* and *x* axes. Consequently, the charge accumulations created on the surface of *y*-cut Fe:LiNbO_3_ by illumination are significantly inhomogeneous in the plane, thus limiting the possible geometries of virtual electrodes that can be realized on these substrates.^19^ On the contrary, the use of *z*-cut substrates allows for the realization of manipulation patterns with arbitrary shapes and ensures enhanced performance of the final device. In particular, the splitting of much larger water droplets (Ω = 3 μL) was achieved by illuminating the *z*-cut Fe:LiNbO_3_ crystal covered with a slippery coating with a circular spot located near the droplet.^32^ However, these initial measurements indicated a fairly unpredictable process, with a splitting success rate of well below 50%.

In this work, we present a comprehensive experimental and theoretical study of the splitting of water droplets on *z*-cut Fe:LiNbO_3_ crystals coated with a slippery lubricated layer and the consequent movement of the fragments along three directions separated by 120°, which reflect the internal symmetry of the crystals induced by the laser beam heating. The presence of the lubricant coating is found to be essential to guarantee excellent droplet splitting reproducibility. Otherwise, contact line pinning effects cause severe limitations to current light-driven technologies of liquid droplets.

## Results

Before analyzing the splitting results, we discuss the initial tests aimed at identifying the best conditions to produce robust lubricated coatings that guarantee very weak pinning of the water droplets, which is a crucial aspect for the reproducible observation of droplet splitting.

### Determination of the Optimal Oil Thickness

**Figure 1:**
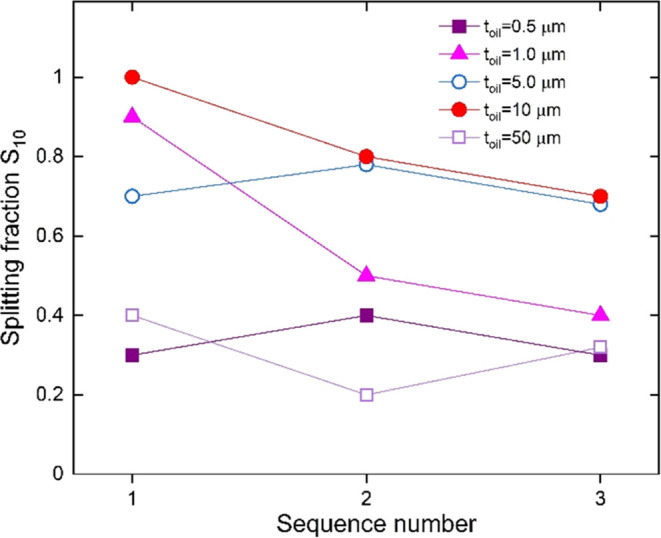
Splitting fraction as a function of the test sequence index for different oil thicknesses. The S10 fraction indicates the number of splitting events that occur in 10 consecutive trials initialized in the same way. In this preliminary study, the intensity of light is *I* = 19.1 kW/m^2^, the droplets have volume Ω = 3 μL, and the circular light spot has a diameter *D* = 3 mm. The oil thickness is varied by changing the extraction speed of the Fe:LiNbO_3_ substrate during dip coating infusion.

Our preliminary results^32^ have shown that it is possible to split a droplet by illuminating a *z*-cut Fe:LiNbO_3_ crystal with a single light spot, although the reproducibility was not satisfactory: the splitting success rate was well below 50% and, once the droplet split, the fragments seemingly followed random trajectories. Therefore, we have systematically investigated possible causes and determined that the quality of the oil layer plays an important role, as is clearly shown in the graph of Figure 1. These measurements refer to droplets of volume Ω = 3 μL, with the contour touching the light spot. Right after droplet deposition, a circular spot with a diameter *D* = 3 mm is illuminated by the laser with a uniform intensity *I* = 19.1 kW/m^2^. The size of the light spot is comparable to that of the sessile droplets: the roles that the size and shape of the light spot play in the splitting process will be the subject of a different study. The trajectory followed by the droplet interacting with the photovoltaic field is recorded at a frame rate of 20 Hz. During the motion, the droplet may split into two fragments or not. Supporting Video S1 shows some representative splitting events obtained by depositing the droplets at different positions around the illuminated contour. The vertical axis in Figure 1 represents the splitting fraction *S*_10_, which indicates the number of splitting events occurring over ten consecutive trials prepared following the same conditions mentioned above. Once a trial is over, the crystal is translated by at least one cm to make sure that the newly exposed surface is not charged, and the whole process is repeated. At the completion of the tenth trial, the crystal is fully discharged by spraying some water on the active surface. Once the surface is dry, a new sequence of ten trials is performed, with the number reported on the horizontal axis representing the corresponding index. The graph shows that, on a freshly prepared surface, all droplets split (*S*_10_ = 1) on an oil film with an estimated thickness *t*_oil_ ≅ 10 μm, while the fraction *S*_10_ drops to about 0.3 on an oil film with *t*_oil_ ≅ 0.5 μm. Discharging the Fe:LiNbO_3_ crystal and repeating the splitting measurements do not appear to dramatically affect these results. From this preliminary study, we find that the optimal choice to obtain reproducible splitting events is *t*_oil_ ≅ 10 μm, which is the nominal value used throughout this study. On much thinner oil films, the droplet motion is likely perturbated by the asperities of the underlying filter, while in much thicker thicknesses, the droplet is immersed in an oil sea and its mobility is severely hampered by the oil ridge/meniscus surrounding the droplet.

**Figure 2:**
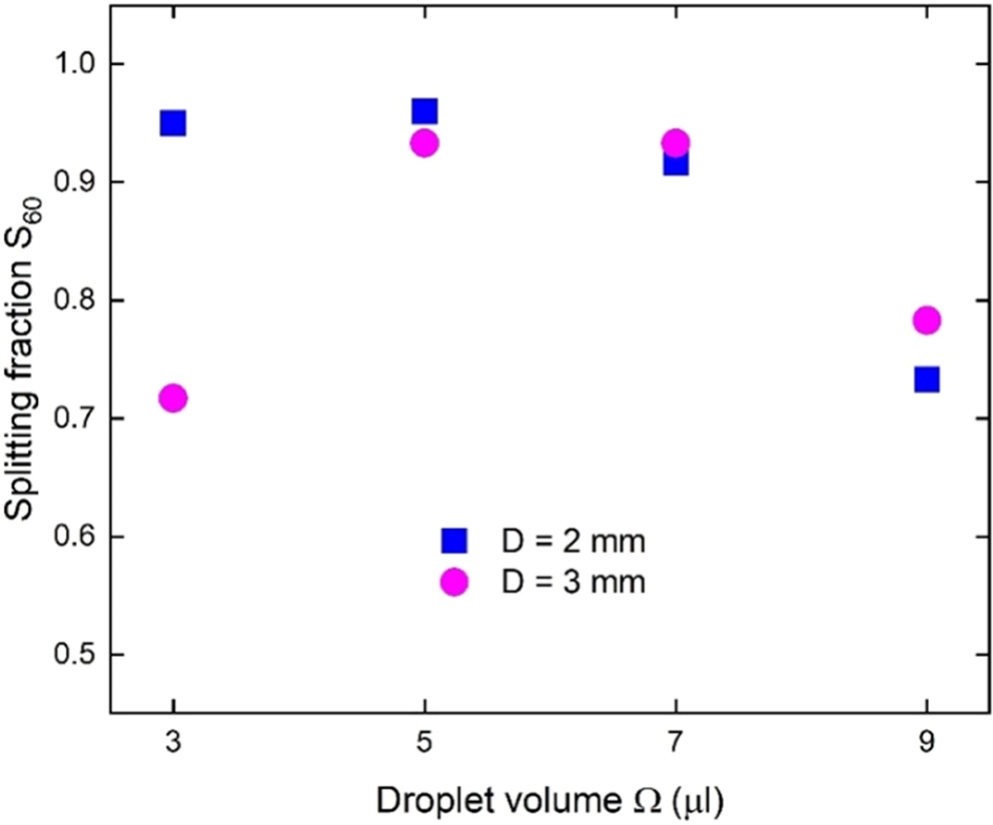
Splitting fraction as a function of droplet volume Ω for different light spot diameters *D*. The *S*_60_ fraction indicates the number of splitting events that occur in 60 consecutive trials prepared under the same experimental conditions. The light spot is illuminated with uniform intensities of *I* = 19.1 kW/m^2^ (*D* = 3 mm) and *I* = 43 kW/m^2^ (*D* = 2 mm). The nominal value of the oil thickness is *t*_oil_ = 10 μm.

Once the optimal oil thickness is identified, a systematic study is carried out to accumulate robust statistics to determine the best configuration to obtain splitting events in a reproducible way. It is based on evaluating the splitting fraction as a function of two main experimental parameters: the volume Ω of the water droplet and the diameter *D* of the light spot. Droplets are initially deposited in four distinct positions along the *x* and *y* axes: above, below, left, and right, with the droplet contour always touching the circular spot. For each position, the evolution of 15 consecutive droplets, prepared following the same experimental procedure described in the Materials and Methods Section, is analyzed. Since no significant differences are observed among these sequences, in the graph of Figure 2, we plot the measured splitting rate of all 60 droplets *S*_60_ as a function of Ω for two different *D*. The data clearly show that for Ω ≤ 5 μL, practically, all droplets split when exposed to the photovoltaic field produced by a light spot with *D* = 2 mm, regardless of the initial position of the droplets.

### Trifurcated Splitting

**Figure 3:**
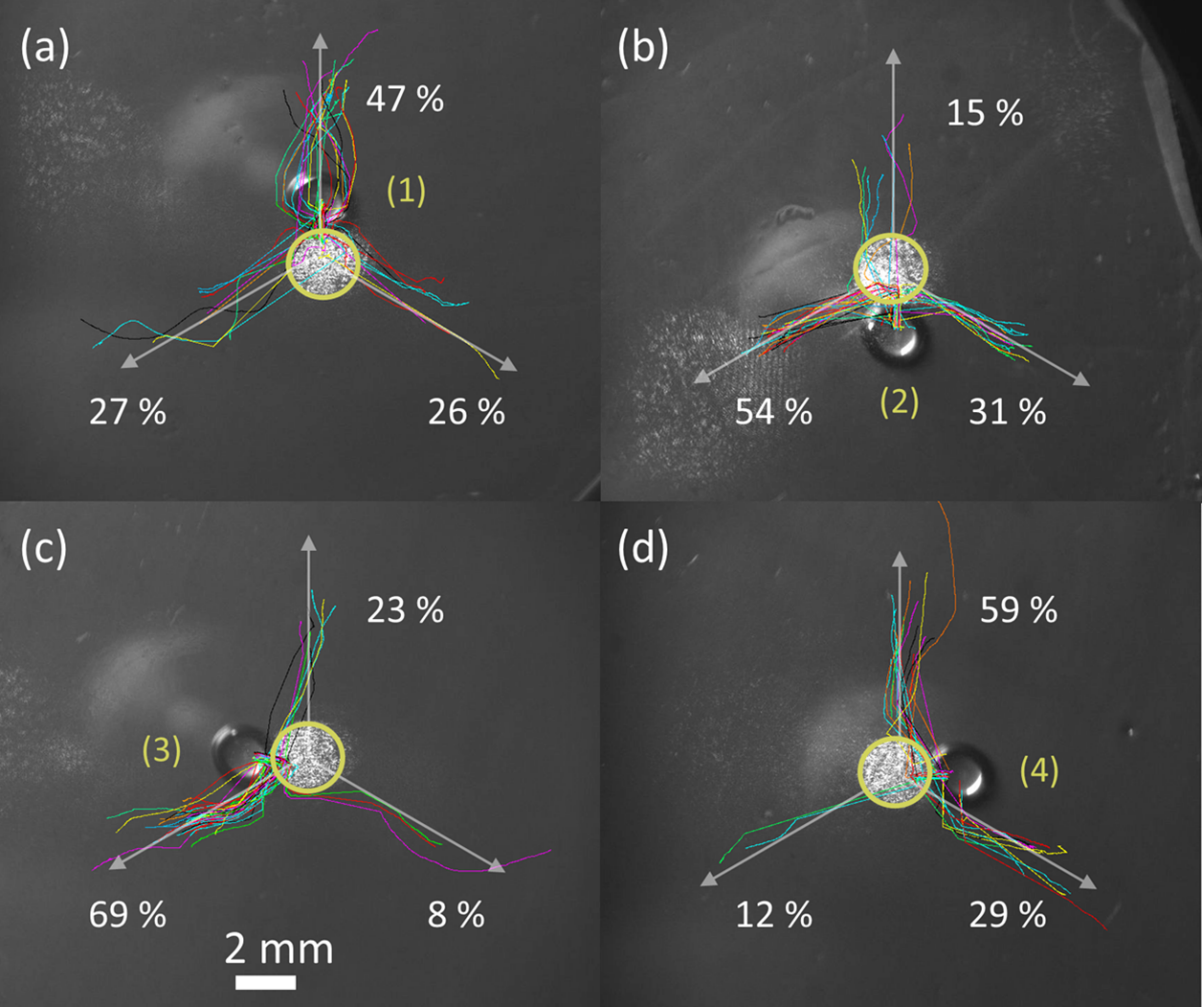
(a–d) Superposition of the trajectories followed by water droplets of volume Ω = 3 μL after the switching on of a circular light spot of diameter *D* = 2 mm and uniform intensity *I* = 43 kW/m^2^. The four panels refer to the different initial droplet positions oriented along the *x* and *y* axes. The numbers (1–4) mark the initial positions of the droplets with respect to the light spot: (1) above, (2) below, (3) left, and (4) right. The yellow circles indicate the contours of the light spots and the white arrows indicate the *y* axes of the Fe:LiNbO_3_substrate. Different colors distinguish the trajectories followed by different droplets. The numbers beside each bundle of trajectories represent the percentage of charged fragments that, after droplet splitting, follow that direction.

Due to this high reproducibility, it is possible to recognize a precise pattern in the directions of splitting. Figure 3 reports the trajectories derived from the analysis of more than 150 videos showing droplets with Ω = 3 μL splitting into two fragments by illumination of a light spot of *D* = 2 mm with uniform intensity *I* = 43 kW/m^2^, see also Supporting Video S2. These curves represent the time evolution of the center of the wetted droplet area. After an initial transient, where the droplet is attracted toward the bright spot, it starts to elongate and finally splits, one fragment being trapped by the bright spot, while the other moves away from it. The elongation is due to the evanescent light-induced field, which has nonzero components parallel to the crystal surface and displaces the positive and negative ions, which are always present in water because of natural ionization. This electrostatic interaction may lead to the breakup of the droplet in a positively charged fragment attracted to the bright laser spot, while the negatively charged fragment is repelled from it. Indirect evidence of the formation of charged fragments can also be found in previous studies involving sessile water droplets on illuminated Fe:LiNbO_3_ crystals.^32,42,43^ A somewhat similar phenomenology is observed in the case of water droplets immersed in oil and subject to high electric fields generated by applying a voltage between two metallic electrodes.^44^ However, the resulting droplet deformation and breakup are highly complex: they depend not only on the strength of the applied electric field but also on physical parameters, such as the properties of the two liquids in contact, the orientation of the droplets in the electric field, and the uniformity of the electric field relative to the droplet.^45^ A rich scenario is found, which includes the splitting of a droplet into two relatively large daughter droplets and several tiny satellite droplets^46^ and the emission of either a stream of droplets or a fine jet that subsequently breaks up into droplets from the conical shape assumed from the droplet.^44,47^ In particular, in the case of individual water droplets settled at the bottom of a plastic box filled with oil and subjected to a high lateral electric field, sometimes a high deformation of the mother droplet is observed along the direction of the electric field followed by its splitting into two or more daughter droplets of similar volume.^48^ Each of these droplets has a charge opposite to that of the adjacent electrodes, causing them to move toward those electrodes.

The trajectory followed by a droplet after splitting does depend on its initial position, as indicated by the percentage values reported beside each bundle of trajectories in Figure 3. These values indicate the fraction of droplets that move in that direction out of 150 repeated trials. For example, when the droplet is placed above the light spot, see position 1 in Figure 3a, it will move up vertically almost half of the time after splitting, while it will equally follow one of the two other directions. Surprisingly, when the droplet is placed below the light spot, see position 2 in Figure 3b, the percentage values vary significantly from one direction to the other. This is probably an artifact due to poor statistics: the percentages are extracted from samples of typically 150 events. Slight asymmetries in the light profile may also contribute. Notwithstanding, these results show that after splitting, the preferred trajectory is the closest to the droplet’s initial position. Regardless of the initial position of the droplet, Figure 3 shows that the negatively charged fragment does not move randomly but rather spontaneously follows one of three well-defined trajectories separated by 120°, reflecting the anisotropic crystalline structure of Fe:LiNbO_3_. This trifurcation recalls that recently reported on the self-propulsion of cold water droplets deposited on a lubricated *z*-cut Fe:LiNbO_3_ crystal.^36^ In this study, after a cold droplet touches the substrate kept at room temperature, it locally cools it down. Such localized cooling propagates, inducing inhomogeneous thermoelastic stresses through the crystal thickness, because of the noncentral symmetric crystal structure. As a result, spatially inhomogeneous surface bound charges are generated, giving rise to a surface potential with one centered negative pole surrounded by three positive poles in 3-fold rotational symmetry. The trifurcated self-propulsion directions of the water droplets are found to match this electric potential pattern.^36^

**Figure 4:**
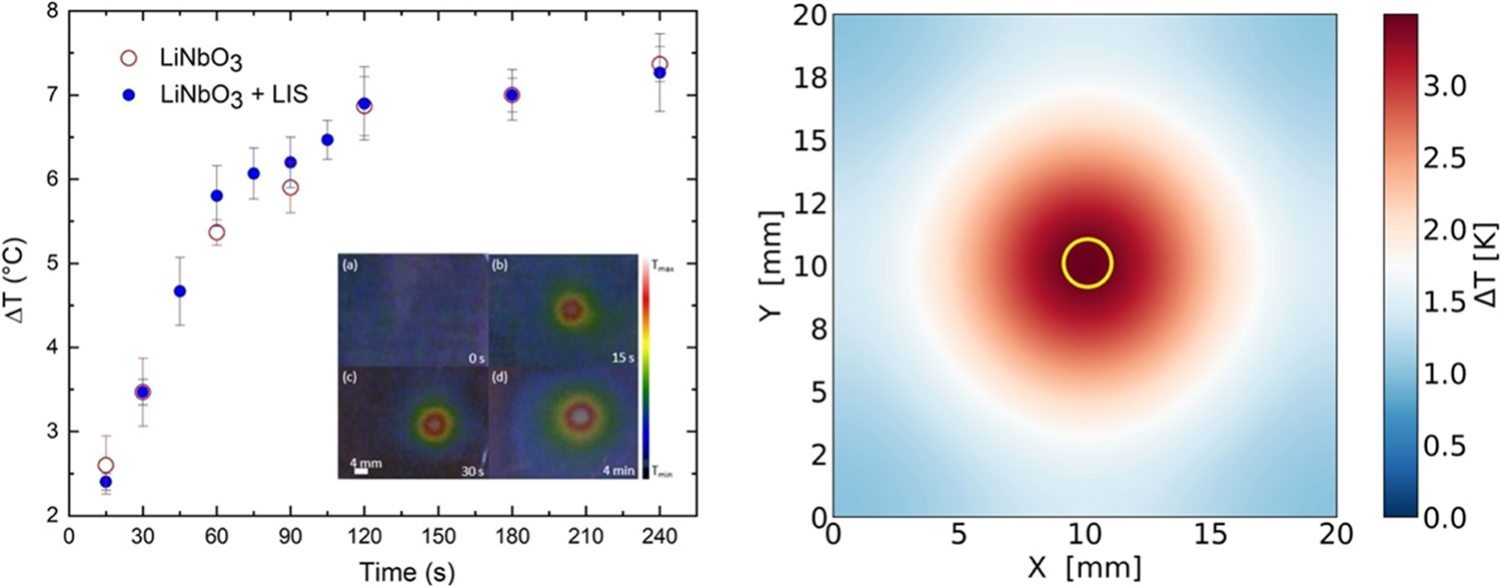
Left: Temperature increase Δ*T* of the Fe:LiNbO_3_ surface measured on a circular spot with *D* = 2 mm and *I* = 43 kW/m^2^ at increasing illumination times. Each data point refers to the mean of at least four different measurements acquired on different days to ensure the reproducibility of the phenomenon. Error bars represent the standard deviation. The inset shows four images of the crystal surface taken with a thermal camera before (a) and after 15 s (b), 30 s (c), and 4 min (d) of illumination. The white cores correspond to the hottest areas of the sample. In (a), the LiNbO_3_ crystal is at ambient temperature around *T* = 23 °C; in (b), the largest temperature difference Δ*T* is ∼ 2.5 °C and the hottest region has a diameter *D* ∼ 2.2 mm; in (c), Δ*T* ∼ 3.5 °C and *D* ∼ 3.0 mm, and in (d), Δ*T* ∼ 7 °C and *D* ∼ 5 mm. Right: Simulated temperature profile along the top face of the Fe:LiNbO_3_ crystal after 30 s of illumination and 2.5 s of laser off.

Thus, we have measured the temperature on the surface of the lithium niobate crystal in correspondence with the light spot. Figure 4 shows the increase in temperature Δ*T* with respect to the initial crystal temperature after illumination of the circular spot that has the same characteristics as those used in the splitting experiments reported in Figure 3, namely, *I* = 43 kW/m^2^ and *D* = 2 mm. The temperature is measured using an infrared camera. Images are taken after illuminating the crystal for a specific time interval, as shown in the figure inset, followed by a time of about 2–3 s required to take the image with the laser off. Image (a), taken before illuminating the crystal, shows a uniform temperature profile across the surface, whose value coincides with room temperature; image (b), taken after 15 s of illumination, shows a marked temperature increase Δ*T* in correspondence with the light spot of about 2.5 °C; image (c) shows that the central core becomes hotter, Δ*T* ∼ 3.5 °C, and wider, *D* ∼ 3.0 mm; finally, after 4 min, the core presents a Δ*T* ∼ 7 °C and widens to *D* ∼ 5 mm due to heat diffusion as shown in image (d). Substantial differences are not found between the bare Fe:LiNbO_3_ crystal and that covered with the LIS, suggesting that heating occurs mainly in the bulk doped crystal and is not affected by the presence of the lubricated coating. This is confirmed by taking images of a common glass slide under the same illumination conditions, which clearly show no temperature variations in the illuminated area. Repeating these measurements with a thermocouple yields the same scenario. These results are confirmed by numerical simulations performed as indicated in the Materials and Methods Section. In particular, the map in Figure 4 shows the calculated temperature profile after illuminating the crystal with a light beam of intensity *I* = 43 kW/m^2^ for 30 s and then turning off the laser for 2.5 s: the core shows a Δ*T* ∼ 3.5 °C, which is in very good agreement with the value measured with the infrared camera. This result can be taken as an implicit validation of the numerical analysis. We expect that the temperature increase of the water droplets will be somewhat less than Δ*T* due to the poor thermal contact with the substrate and the negligible optical absorbance of water. In other words, the increase in the droplet temperature in response to light illumination is not critical for the possible biochemical applications of this platform.

### Numerical Calculations

To analyze the observed behavior of droplet motion, we developed a numerical code (described later in the Materials and Methods Section) to calculate the evanescent electric field in proximity to the slab surface. We implemented an option to set the temperature and photovoltaic charge distributions in a realistic manner. The code accounts for all of the pyroelectric, piezoelectric, and photovoltaic effects. Finally, the code can calculate, in addition to the electric field inside and outside the slab, the dielectrophoretic force acting on a spherical droplet on the slab surface. Approximating the droplet as a regular sphere makes the estimate of forces feasible.^43^ Work is in progress to include in numerical investigations a realistic simulation of droplets with optimized geometries.^49^ It is worth noting that in the limit of a high relative dielectric constant, as in the case of water, the forces on a dielectric sphere and on a conducting one practically coincide.

**Figure 5:**
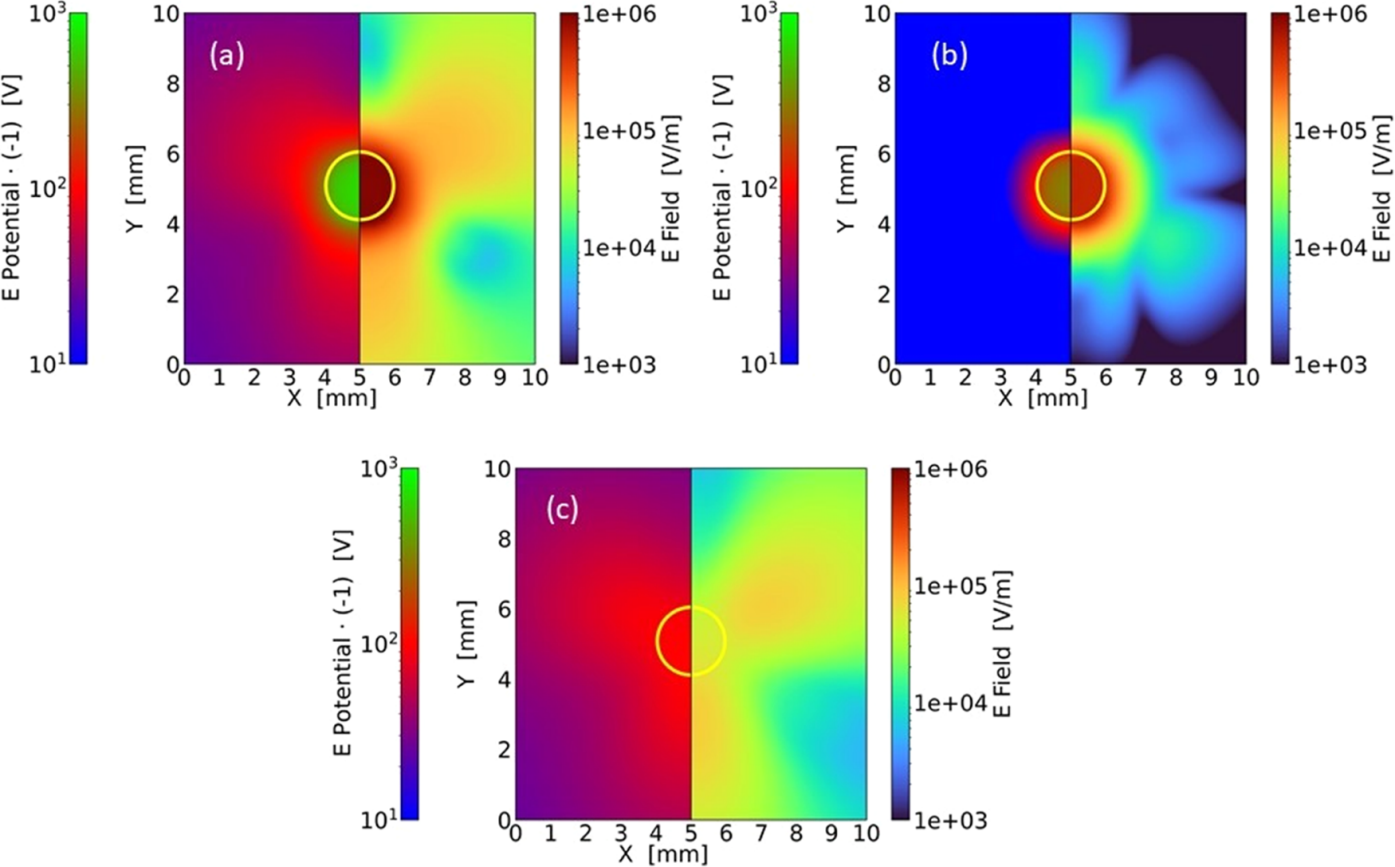
Magnitude of the electric field (right halves) and the electric potential change of sign (left halves) calculated on the upper surface, *z* = 0, of a *z*-cut Fe:LiNbO_3_ crystal. Yellow circles indicate the contour of a circular disk with a diameter *D* = 2 mm. Panel (a) refers to the actual experimental conditions: surface charge density of the illuminated disk σ_c_ = 720 μC/m^2^ and temperature increase relative to the nonilluminated area Δ*T* = 7 °C. Panel (b) shows the electric contributions due to the photovoltaic effect alone: σ_c_ = 720 μC/m^2^ and Δ*T* = 0 °C, while panel (c) reports that caused only by the pyroelectric effect: σ_c_ = 0 μC/m^2^ and Δ*T* = 7 °C.

We first performed a simulation accounting for the same conditions of temperature and surface charge accumulation because of the photovoltaic effect, as observed in the experiment. In panels (a–c) of Figure 5, we report the magnitude of the evanescent field and the electric potential, both evaluated at the top surface of the crystal. We note that the potential shows the same 3-fold symmetry, as reported by Tang et al.,^36^ for a cold droplet deposited on the surface of a *z*-cut Fe:LiNbO_3_. The maximum potential difference of about 400 V calculated near the contour of the illuminated circle is a factor of 2 greater than that close to a cold droplet that has a temperature of −18 °C with respect to the crystal.^36^ The electric field of Figure 5a presents the same symmetry, although with a more complex pattern, and reaches its maximum magnitude, about 5 × 10^5^ V/m, in correspondence with the illuminated spot. Interestingly, the magnitude of the electric field outside this spot remains quite high, about 1.5 × 10^5^ V/m, in the lobes.

These results are due to a combination of pyroelectric, piezoelectric, and photovoltaic effects. We then performed two additional simulations to disentangle the role of the temperature from that of the photoinduced charges. Figure 5b shows the electric field and potential generated by a charged disk assuming a uniform temperature distribution in the crystal, that is, no local heating due to the illumination but only photoinduced charging. As expected, the resulting potential mainly exhibits circular symmetry in correspondence with the light spot, with values of the electric field of up to 10^6^ V/m. The 6-fold symmetry of the electric field around the circular spot is due to the photoinduced charge transport occurring in the horizontal plane; anyway, the in-plane electric fields are an order of magnitude lower with respect to the one along the *z* axis, as expected by the photovoltaic matrix for LiNbO_3_ (see the Materials and MethodsSection). Moreover, the values of the photoinduced electric field magnitude are smaller than those calculated by Muñoz-Martínez et al. for a *z*-cut Fe:LiNbO_3_ crystal^24^ due to both the different iron concentrations of their LiNbO_3_ crystals and the use in our work of values for photovoltaic parameters that have recently been derived taking into account the small polaron contribution to the charge transport model.^50^ Finally, Figure 5c reports the electric field and potential caused by a cylindrical portion of the crystal that has a uniform temperature of 7 °C higher than that of the surrounding material, assuming that there are no photoinduced surface charges. The trifurcated symmetry is clearly recovered, although the electric field and potential are now significantly lower than those in the initial case.

## Discussion

**Figure 6:**
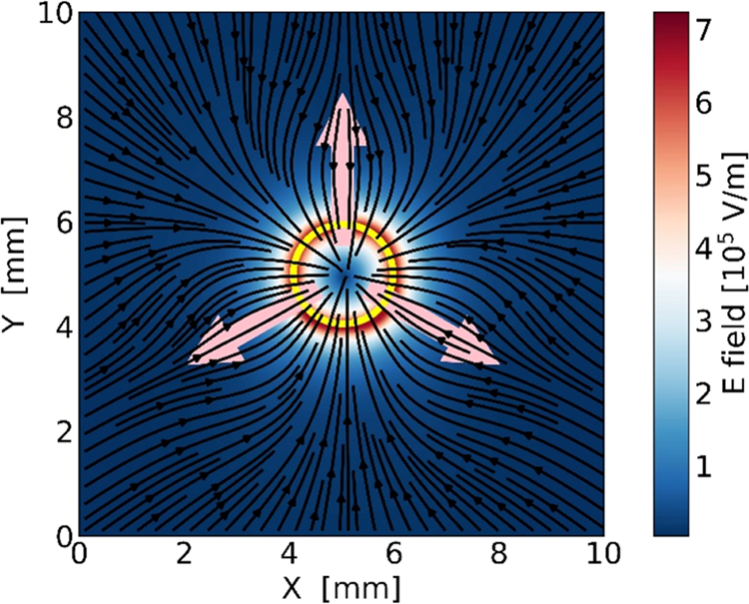
Magnitude of the projection of the electric vector field on the horizontal plane calculated on the top layer of the slab, together with the electric field flow lines. The three arrows are pointed in the direction of the three planar axes; the yellow circle highlights the contour of the illuminated spot.

The observed splitting and motion of the droplets can be understood by considering the evanescent field generated by illuminating the Fe:LiNbO_3_ crystal in Figure 5a. To this regard, Figure 6 displays the planar component of the evanescent electric field vectors and the corresponding flow lines calculated on the top surface of the crystal, *z* = 0, obtained by numerical integration of the equations, describing the involved multiphysics processes as outlined before. It clearly shows that the vectors are oriented toward the center of the illuminated spot and their amplitude exceeds 7 × 10^5^ V/m up to about 1 mm from the edge of the spot. These high values are comparable to those obtained in the droplet breakup of water droplets immersed in oil.^44−46^ In particular, the strength of the applied direct current (DC) electric field required to split a 1 mm diameter water droplet sitting in a plastic box full of oil is found to be around 3 × 10^5^ V/m, where the average electric field strength is taken as the ratio of the applied potential difference to the separation distance between the two electrodes.^48^ A larger initial droplet will require a lower electric field strength to cause breakup.^46^ Interestingly, the amplitude of the evanescent field due to the pyroelectric effect alone increases in the correspondence of the three symmetric lobes and reaches a maximum of approximately 1 × 10^5^ V/m in correspondence to the heated spot, as shown in Figure 5c. However, this field is 10 times smaller than the value calculated at the same point because of the combination of the pyroelectric and photovoltaic fields displayed in Figure 5a. More importantly, it is well below the critical field required for droplet splitting, and this explains why, in the experiments by Tang et al. on the furcated motion of cold droplets on crystals,^36^ no splitting is reported.

**Figure 7:**
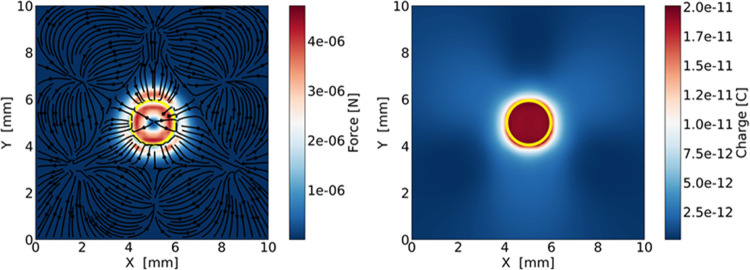
Left panel: Planar projection of the dielectrophoretic force for an ideal spherical water droplet placed on top of the slab. The graph reports the dielectrophoretic force acting on the droplet with respect to the position of the center of the droplet. Right panel: Polarization charge as a function of the position of the center of the droplet at the moment of splitting. The yellow circle indicates the contour of a circular disk of diameter *D* = 2 mm, which corresponds to the illuminated area.

The motion of a neutral droplet on the slab toward the laser spot is due to two processes. First, a dipole moment is generated on the droplet in response to the electric field. As the electric field in Figure 6 is directed toward the center of the spot, the surface of the droplet oriented toward it will become positively charged. Then, an electric force will be exerted on the positive and negative charges induced on the droplet. As the electric field is not homogeneous, the two contributions do not compensate for each other, and a net dielectrophoretic force appears. In Figure 7a, we show the planar projection of the dielectrophoretic force for a spherical droplet of Ω = 3 μL, in the vicinity of the laser spot. The force points toward the center of the spot and its magnitude reaches 3 μN, which is comparable to the static friction of droplets of the same volume deposited on the lubricated surface, which is 3 ± 0.5 μN. We derived this value by measuring the sliding angle, that is, the minimum angle of inclination of the lubricated surface above which the droplet begins to move,^51^ with the laser off.

Interestingly, when the center of the droplet is inside the laser spot, the force changes direction; see Figure 7a. This is directly related to the decrease in the magnitude of the in-plane electric field inside the laser spot, as shown in Figure 6, and this is in line with the observed motion of the water droplets. Indeed, from the Supporting Videos S1 and S2, it can be clearly seen that the neutral droplet does not reach the center of the spot but stops and splits well before. Here, the change in the external evanescent electric potential throughout a droplet of Ω = 3 μL is a few hundred volts, enough to split the droplet.^52^ When a water spherical drop is placed in an electric field, positive charges are induced on one side of the surface and negative charges are induced on the other. Consequently, when the main droplet splits, the resulting two droplets present a non-null charge, whose absolute value depends on the position of the main droplet at the moment of the splitting with respect to the illuminated area. This dependence is clearly visible in Figure 7b, which displays the polarization charge, that is, the charge accumulated in the positive and negative halves of the surface, for the main droplet as a function of the position of its center. Breaking of the droplet separates the two domains so that the two fragment droplets acquire a net charge. From Figure 7b, it is possible to observe that the maximum charge of the two droplets (i.e., the charge accumulated in the positive and negative domains of the main droplet) is achieved when the splitting phenomenon occurs in proximity or inside the illuminated spot. From the geometry of the system right before splitting, namely, the size of the illuminated spot, the distance of the droplet center from the spot center, and the droplet volume, we infer an absolute maximum charge of about 2 × 10^–11^ C. Therefore, from the electric field reported in Figure 6, we can derive a repulsive Coulomb force of about 10 μN exerted on the negative droplet fragment.

After splitting, the positive fragment is attracted toward the negatively charged illuminated spot and remains trapped at its center. On the contrary, the negative fragment is repelled from the central spot and follows backward the flow lines of Figure 6, which concentrate along the three directions that correspond to the *y* axes of the *z*-cut Fe:LiNbO_3_ crystal (indicated with pink arrows), producing the trifurcated motion shown in Figure 3.

We point out that the calculated amplitude of the electric field is not sufficient to explain the splitting and its remarkable reproducibility. Another key ingredient is the presence of a lubricated surface. In fact, if the crystal is coated with a micrometer solid film that exhibits a larger contact line pinning, the splitting rarely occurs. The highly slippery lubricated surface allows the droplet to slowly vary in shape and align its position with respect to the electric field to maximize the electrostatic interaction. After splitting, the relatively small electric force is enough to overcome the static friction force with the surface and drive the fragment along the symmetry axes of the crystal.

## Conclusions

We have presented an optofluidic device capable of splitting millimetric droplets in a reliable way and driving the resulting fragments along well-defined trajectories. It is based on a *z*-cut Fe:LiNbO_3_ substrate coated with a lubricant-infused layer, which guarantees a very slippery surface. Such an engineered surface has recently been shown to provide an easily configurable platform^32^ to drive water droplets in a controllable way through light beams, but not to split droplets. For this specific task, *y*-cut Fe:LiNbO_3_ is generally employed^28,40^ due to the photoinduced generation of an evanescent field oriented primarily parallel to the main surface rather than orthogonally to it, as in a *z*-cut crystal. In contrast, in this work, we have shown that the employment of *z*-cut Fe:LiNbO_3_ crystals also ensures the splitting of water droplets. This phenomenon has also been investigated in detail by performing dedicated simulations, which highlighted the role played by the dielectrophoretic force both in governing the motion of the water droplets toward the illuminated spot and in achieving the final splitting. In particular, the numerical analysis suggests that the splitting process is essentially caused by the strong electric field produced close to the illuminated spot due to the photovoltaic effect, which can easily overcome the critical field of approximately 3 × 10^5^ V/m required to break a millimetric droplet.^48^ Instead, the pyroelectric effect caused by the heating of the crystal after illumination is responsible for the directional motion of the fragments along the three symmetry axes of the *z*-cut Fe:LiNbO_3_ crystal. Using crystals of different symmetry can change these directions in a controllable way. Regardless of the nature of the crystal, to obtain reproducible results, it is essential to coat the photoactive substrate with a lubricated film of controlled thickness to reduce the surface pinning of the droplets.

This study suggests a promising synergy between the light illumination required to generate the photovoltaic field and the pyroelectric effect caused by partial absorption of light, which introduces spatial asymmetries in the overall electric field. The trifurcated directions are not imposed by external, complicated fabrication constraints such as functionalization of chemical inhomogeneities or texture of morphological anisotropies on the substrate but depend only on the specific cut of the photoactive crystal. In our case, we used a *z*-cut Fe:LiNbO_3_ crystal that induces three directions equally spaced by 120°, but unidirectional, bidirectional, and other configurations are possible for different cuts and crystals.^36^ Furthermore, the relative weights of the photovoltaic and pyroelectric effects can be tuned by playing with the degree of reduction of the crystal. Overall, this synergy can provide a valuable feature in applications that require the splitting and coalescence of droplets, such as DNA library preparation, immunoassay optimization, and nucleic acid. More generally, these results confirm that the optofluidic platform^32^ based on a lubricated *z*-cut Fe:LiNbO_3_ crystal is a viable alternative to microfluidic electrowetting devices:^53^ on such a photoactive substrate, millimetric droplets can be actuated, guided, merged, and split in a flexible and reconfigurable manner simply by illuminating the crystal, without the use of moving parts or the need for cumbersome electrical connections of electrowetting devices. The characteristic times of a few seconds required to move or split a droplet do not represent a limitation for applications in bioanalytical assays.

## Materials and Methods

### Iron-Doped Lithium Niobate Crystals

The experiments have been performed using a single-domain iron-doped lithium niobate crystal (Fe:LiNbO_3_), supplied by PI-KEM Limited. The iron concentration has been chosen equal to 18.8 × 10^24^ at/m^3^ (0.1% mol), to guarantee optimal performance in terms of the amplitude of the photovoltaic field.^54,55^ The degree of reduction degree *R* = [Fe^2+^]/[Fe^3+^] plays a key role in determining the photovoltaic response of the material; therefore, the amounts of donor (Fe^2+^, *N*_D_) and acceptor (Fe^3+^, *N*_A_) ions have been determined by optical absorption measurements^56^ using a Jasco V-670 spectrophotometer, resulting in *R* = 0.32 ± 0.01 (*N*_D_ = 4.6 × 10^24^ at/m^3^, *N*_A_ = 14.2 × 10^24^ at/m^3^).

**Figure 8:**
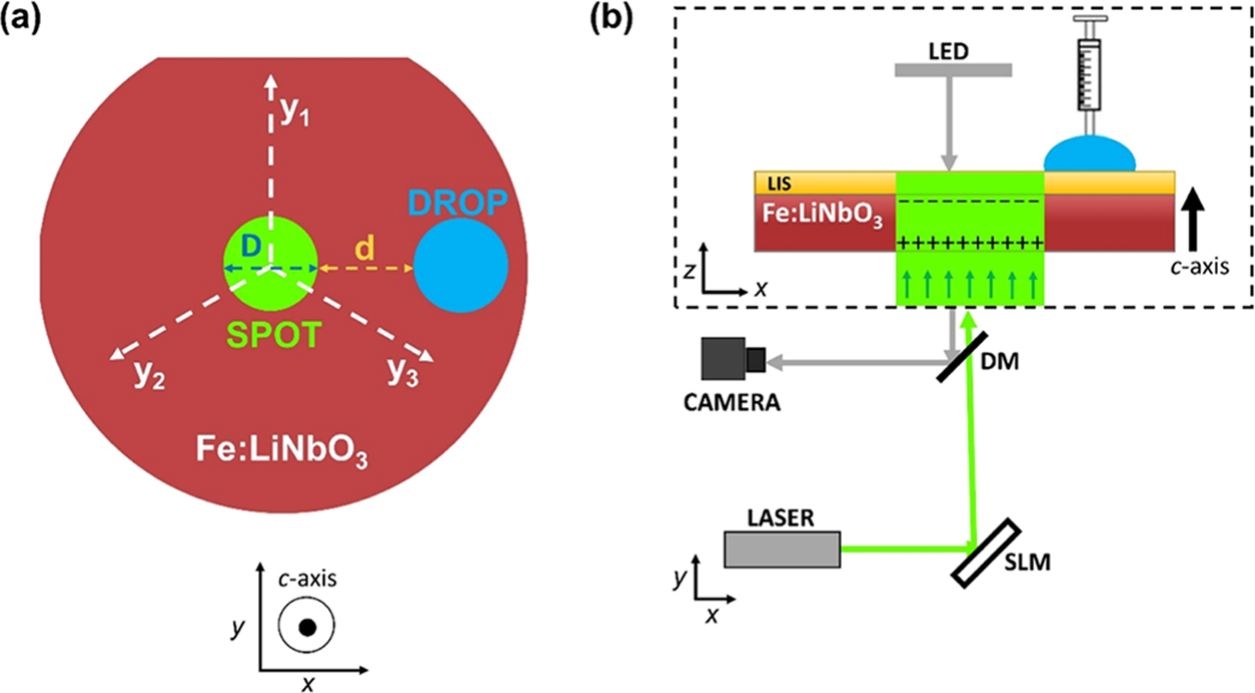
(a) Top view of the Fe:LiNbO_3_ crystal showing the circular light spot in the center and a water droplet to its right. The dashed lines represent the *y* axes of the substrate, whereas the main faces of the wafer are perpendicular to the *z* axis (*c* axis) of the material. (b) Top: Lateral view of the Fe:LiNbO_3_ crystal showing photoinduced surface charging and the deposition of a water droplet with a syringe pump. Bottom: Schematic of the optical path followed by the laser beam to project a light pattern on the Fe:LiNbO_3_ crystal coated with a lubricant-infused surface (LIS). The laser beam is expanded to fit the area of the spatial light modulator SLM, and the resulting light pattern illuminates the Fe:LiNbO_3_ crystal perpendicular to the bottom. DM is a dichroic mirror used to observe the displacements of the droplet. See the main text for further details.

The main faces of the crystal are perpendicular to the polar *c* axis of the material (*z*-cut or (0001) Fe:LiNbO_3_ crystal), and the directions of the in-plane *y* and *x* axes are determined by a high-resolution X-ray diffraction (HR-XRD) technique. The characterization is carried out using a Philips X’Pert PRO MRD diffractometer equipped with a Cu anode source (Cu k_αII_ radiation emission at λ = 0.154 nm), exploiting the asymmetric reflections (336). The larger flat shape of the Fe:LiNbO_3_ wafer results perpendicular to the *y* axis, which lies in the mirror plane of the material. Since lithium niobate belongs to the *R*3*c* space group, the three *y* axes are mutually oriented by 120°, as depicted in Figure 8a.

### Optofluidic Setup

The experimental setup has been described elsewhere^32^ and is shown schematically in Figure 8b. The hybrid substrate is mounted horizontally on a motorized *x*–*y* table (M-126CG, PI) equipped with a customized rotation stage, which allows for in-plane rotations. A circular light spot of diameter *D* and uniform intensity *I* is projected onto the Fe:LiNbO_3_ crystal (for more technical details, see ref (32)). The laser source (Azur Light Systems ALS-GR-532–1-I-SF, maximum output power of 1 W ± 0.5%, λ = 532 nm) is kept at constant output power for optimal stability. The linearly polarized laser beam is attenuated and expanded before being collimated toward the spatial light modulator (SLM, Pluto-NIR-011, Holoeye Photonics). The resulting light pattern is a light spot with a uniform intensity profile, which illuminates the Fe:LiNbO_3_ crystal perpendicular to its main surfaces from the bottom. Taking into account the losses of the optical components, the power *P* of the light pattern impinging on the sample can vary between 0 and 150 mW, and a computer-controlled mechanical shutter allows control of the exposure time.

Ultrapure water droplets (resistivity 18.2 MΩ·cm) of known volume are generated with a column syringe pump (UltraMicroPump by World Precision Instrument) equipped with a glass syringe (500 μL, SGE), whose needle is placed 2 mm above the crystal surface. In this way, droplets can be produced with a volume between 0.3 and 20 μL and deposited in a well-defined position of the Fe:LiNbO_3_ crystal. The distance *d* between the circular light spot and the droplet contour, see Figure 8a, can be precisely adjusted by moving the syringe with a manual translator stage along the *x*–*y* directions.

The droplets are viewed from the bottom using a CCD camera (Basler acA1300–200 μm), equipped with optical zoom lenses (respectively, Baumer Linos and Navitar MVL 7000). A white LED illuminates the droplet from the top and is first reflected by a mirror fixed below the sample holder and then by a dichroic mirror (MD568, Thorlabs), which lets pass the green light (532 nm) from the laser and reflects the red component of the white led light used to back-illuminate the sample. Finally, an emission filter (MF620–52, Thorlabs) is placed before the camera to remove any residual back reflection of the laser beam. Finally, the determination of the temperature increase caused by light absorption is made with an infrared camera (HT-A1 by Hti).

The acquired videos are analyzed offline with custom programs. The software that tracks the droplet trajectories is written in C++ by using the open source library for computer vision openCV. A threshold filter is applied to each video frame to better identify and track the droplet. Then, the software looks for the point of maximum correspondence with a template of a droplet with a certain confidence level. Over a fixed threshold of confidence, the positions are stored, and the trajectory can be reconstructed.

### Preparation of Liquid-Infused Surfaces (LISs)

For the success of these experiments, it is essential to use highly slippery surfaces. For this study, the LIS is obtained by fixing on the +*z* face of the Fe:LiNbO_3_ crystal, a ∼25 μm thick porous polytetrafluoroethylene (PTFE) membrane (Sterlitech Co.) infused with Fomblin, and a fluorinated oil^57^ with viscosity μ = 120 mPa s at *T* = 20 °C and surface tension γ = 21 mN/m at *T* = 20 °C. A dip coater is used to guarantee controlled and reproducible results.^32^ By changing the extraction speed *V*, it is then possible to vary the thickness of the oil layer that covers the membrane, *t*_oil_, whose value can be estimated from the Landau–Levich–Derjaguin (LLD) equation:^58^*t*_oil_ ≈ 0.94*L*_c_*Ca*^2/3^, where  is the capillary length of the oil and *Ca* = μ*V*/γ is the capillary number. In our study, we have explored *t*_oil_ with nominal values in the range of 0.5–50 μm. The resulting LIS can be used safely for the motion of thousands of droplets, which corresponds to about a week of laboratory use. When the droplets start to pin on the surface, the LIS can be easily regenerated by repeating the dip-coating process, see ref^32^ for more details.

### Continuum Equations of the Fe:LiNbO_3_ Crystal

Lithium niobate is a noncentrosymmetric crystal that exhibits a remarkable bulk photovoltaic effect, which can be enhanced by doping it with multicharge transition metals, such as iron. Specifically, within the LiNbO_3_ matrix, iron is incorporated with two possible valence states, Fe^2+^ and Fe^3+^, which substitute Li ions.^59^ Iron ions give rise to additional energy levels within the energy gap of lithium niobate,^60^ with a broad absorption band presenting a maximum of around 477 nm, which are involved in the transport mechanisms of electrons photoexcited with visible light. In fact, in a Fe:LiNbO_3_ crystal under illumination, electrons are photoexcited from donor ions Fe^2+^ and trapped by acceptor ions Fe^3+^, diffusing mainly along the + direction of the *c* axis due to the photovoltaic effect. The photovoltaic current density vector *J*_*i*_ can be expressed as
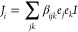
1where *e*_*j*_ and *e*_*k*_ are the unit vectors of the light polarization, *I* is the intensity of the incident light, and β_*ijk*_ is the photovoltaic tensor for linearly polarized light.^59^ The main contribution to the photovoltaic current arises along the *z* axis of the material and is almost independent of light polarization since β_*zxx*_ = β_*zyy*_ and β_*zzz*_ are nearly the same size (4 × 10^–8^ V^–1^); in contrast, the in-plane contributions to the photovoltaic current along the *y* axis are practically reduced to one-tenth, since the elements β_*yxx*_ = β_*yyy*_ are smaller by almost 1 order of magnitude (2 × 10^–9^ V^–1^).^59^ Therefore, photoexcited electrons easily accumulate on the +*z* face of the Fe:LiNbO_3_ crystal, leading to a surface charge density^50^, where ϵ_*zz*_^r^ = 28 is the relative dielectric constant along the *z* axis,^61^ ϵ_0_ the dielectric constant in vacuum, and *L*_pv_ and Λ are the mean photovoltaic transport length and the drift coefficient,^24,50,59^ respectively. According to the values of *L*_pv_ and Λ reported in the literature,^50^ in the sample used in this study, surface charge densities of up to 720 μC/m^2^ are expected to be photoinduced, leading to a photovoltaic field inside the crystal of approximately 3 × 10^6^ V/m. The evanescent field expanding in the space outside the crystal attracts water droplets toward the illuminated spot, as clearly found with the pendant drop method.^62^

Because the lithium niobate crystal is a piezoelectric and pyroelectric material, an internal electric field and a temperature field yield linear contributions to the mechanical stress in addition to the standard elastic contribution due to the strain induced by mechanical displacements *u*(***r***) with respect to the equilibrium configuration. In formal terms, the stress tensor σ_*i,j*_(***r***) in a generic point inside the material indicated by the vector position ***r*** can be expressed as^63^
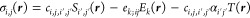
2where *c*_*i*,*j*,*i′*,*j′*_ is the elastic stiffness tensor, *S*_*i*′,*j*′_ (***r***) is the strain rate tensor, *e*_*k*;*ij*_ is the piezoelectric coupling tensor, *E*_*k*_ (***r***) is the photovoltaic field, the minus sign indicates that a negative charge accumulates on the +z face of the Fe:LiNbO_3_ substrate under illumination, α_*i*′*j*′_ is the thermal expansion tensor, *T*(**r**) represents the temperature difference with respect to the equilibrium (room) temperature *T*_0_ and, as before, the minus sign before the temperature term indicates that a negative charge accumulates on the +*z* face of the Fe:LiNbO_3_ substrate under heating, as in the case of intense laser illumination. In reporting equations, we sum over repeated indices.

Similarly, the dependence of the dielectric displacement vector *D* on the photovoltaic field *E*, the strain rate *S*, and the temperature difference field *T* can be expressed as
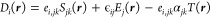
3where ϵ_*ij*_ is the low-frequency dielectric tensor. Finally, the time evolution of the mechanical displacement is given by
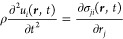
4

### Code Implementation

In the simulations, a *z*-cut Fe:LiNbO_3_ slab of height *h* = 1 mm and edges *L* = 2 cm was considered. To calculate the difference in the temperature difference distribution *T*(***r***) in the slab, the heat diffusion equation in the presence of the heat source due to the laser was explicitly taken into account
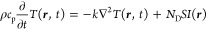
5where *k* = 4.18 W m^–1^ K^–1^ is the thermal conductivity of the slab, ρ = 4650 kg m^–3^ is the mass density, *c*_p_ = 628 J kg^–1^ K^–1^ is the heat capacity at constant pressure, *N*_D_ is the density of electron donors, *S* = 2 × 10^–22^ m^2^ is the photon absorption cross section, and *I*(***r***) is the light intensity inside the slab.^60^ The latter is not zero only in correspondence with the illuminated cylindrical volume of radius *R* = 1 mm and varies along the *z* axis as
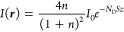
6with *n* = 2.3 is the optical refractive index and *z* is the vertical distance measured from the bottom surface, where the laser beam enters into the crystal with an intensity *I*_0_.

We considered modified Neumann boundary conditions to account for the thermal emission of the slab. These read on slab surfaces
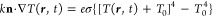
7where **n** is the unit vector normal to the surface, *e* = 0.95 is the relative emissivity of the slab, σ is the Stefan–Boltzmann constant, and *T*_0_ = 298 K is the room temperature, which is equal to the starting temperature of the slab before illumination.

For a given temperature distribution inside the slab, a stationary solution of eq 4 is searched under the constraints of eqs 2 and 3. To find a stationary solution, a friction term is added to eq 4
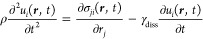
8whose value γ_diss_ = 0.1 kg/(m^3^s) is chosen to maximize the speed and accuracy of the calculation.

To solve eqs 5 and 8, we developed a new Fortran90 code based on finite differences. The slab was sampled using *N*_*x*_ = *N*_*y*_ = 200 grid steps along the two horizontal directions and *N*_*z*_ = 20 steps along the vertical direction. The temperature field was propagated through eq 5 for 30 s, as the experimental delay between laser turning on and droplet deposition, using time steps of 10^–4^ s. The initial displacement field ***u***(*t* = 0) = 0 was propagated through eq 8 using the leapfrog algorithm. The choice of γ_diss_ was dictated by convenience reasons: the stationary state is found to be independent of its precise value. At each time step, the electric potential was obtained by solving the corresponding Poisson equation by using the Richardson algorithm. For this task, two vacuum layers were added above and below the Fe:LiNbO_3_ slab, having the same slab size. These three layers form the simulation cell. The electric potential at the boundaries of the simulation cell and the normal strain component on the surface of the slab were imposed to be null.

Simulations also included the option of adding a circular uniform surface charge distribution of radius *R*, corresponding to the radius of the spot illuminated by the laser, to account for the photovoltaic effect: on the upper (lower) *z*-surface, the surface charge density is negative −σ_c_ (positive +σ_c_). The simulations were run with a time step *t* = 10^–9^ s until a stationary state was reached (i.e., for 5 μs). Finally, the evanescent electric field was calculated from the charge density distribution found, setting the electric potential to zero at an infinite distance.

The electric dipole induced by the evanescent electric field on a spherical droplet with the center at ***r*** and radius *R* is given by^64^
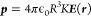
9with *K* = (ϵ – 1)/(ϵ + 2) for a dielectric droplet with a relative dielectric constant ϵ (for water ϵ = 80). For simplicity, we approximated the dipole as two point charges lying in correspondence with the centers of the positive and negative charge distributions on the droplet surface. The absolute value of these charges is as follows
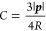
10and the dielectrophoretic force exerted on the droplet is^64^
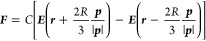
11We checked the convergence of our simulations with respect to the increase in the densities of the applied space–time grids.

## Supporting Information Available

Representative splitting of three droplets initially positioned in different regions around the light spot, showing the motion of the fragments along three directions angularly separated by 120°. Experimental details: volume Ω = 3 μL, power of the light pattern that impinges on the sample *P* = 135 mW, intensity *I* = 43 kW/m^2^, diameter of the light spot *D* = 2 mm (Video S1) (AVI)
Sequence of repeated droplet splittings showing the motion of the fragments along the three *y* axes of the *z*-cut Fe:LiNbO_3_ crystal. The video is shown at twice the normal speed. Experimental details: Ω = 3 μL, intensity *I* = 43 kW/m^2^, diameter of the light spot *D* = 2 mm (Video S2) (MP4)

## Supplementary Material

am3c16573_si_001.avi
